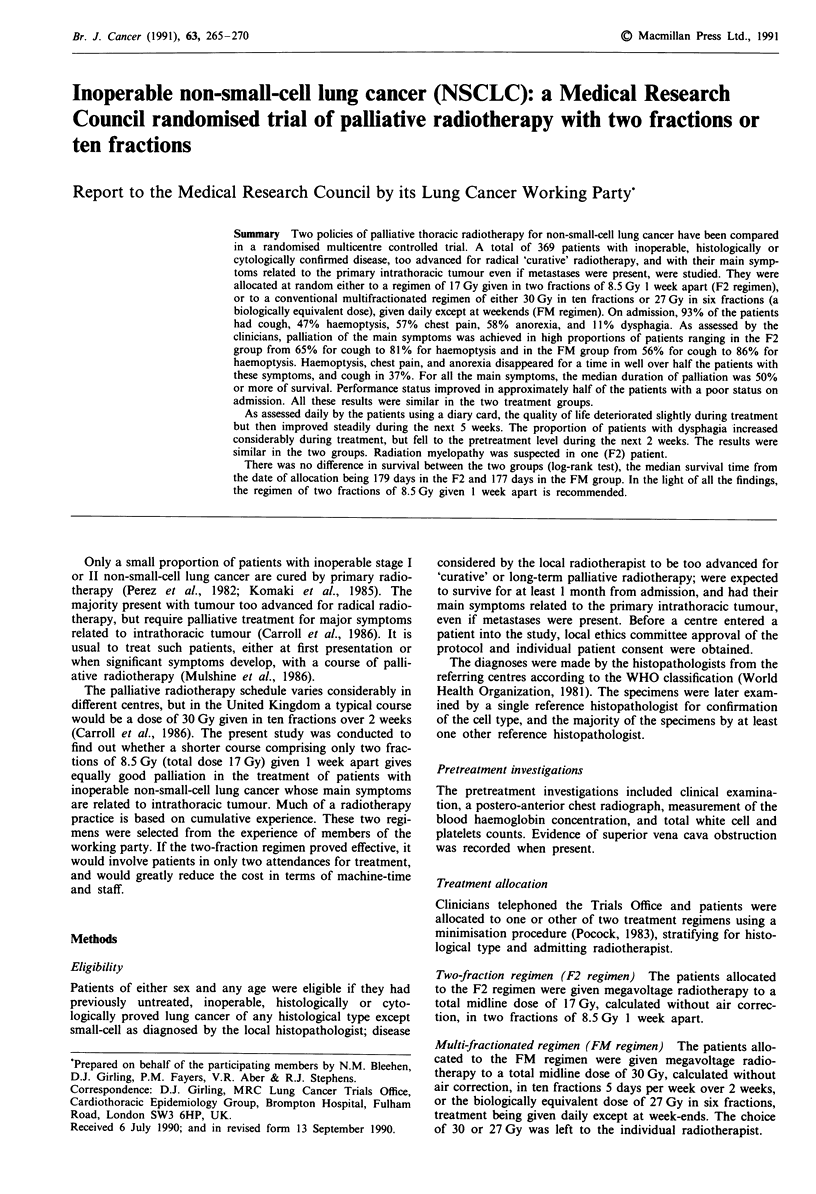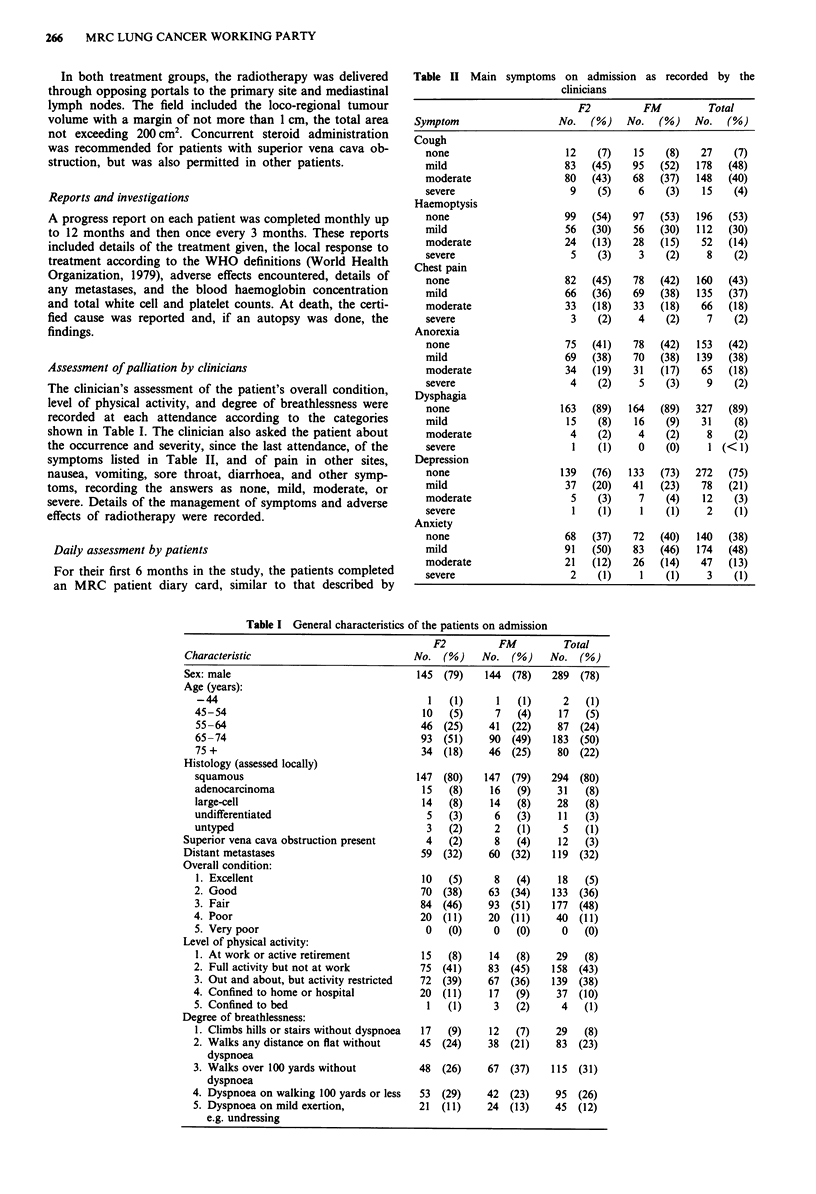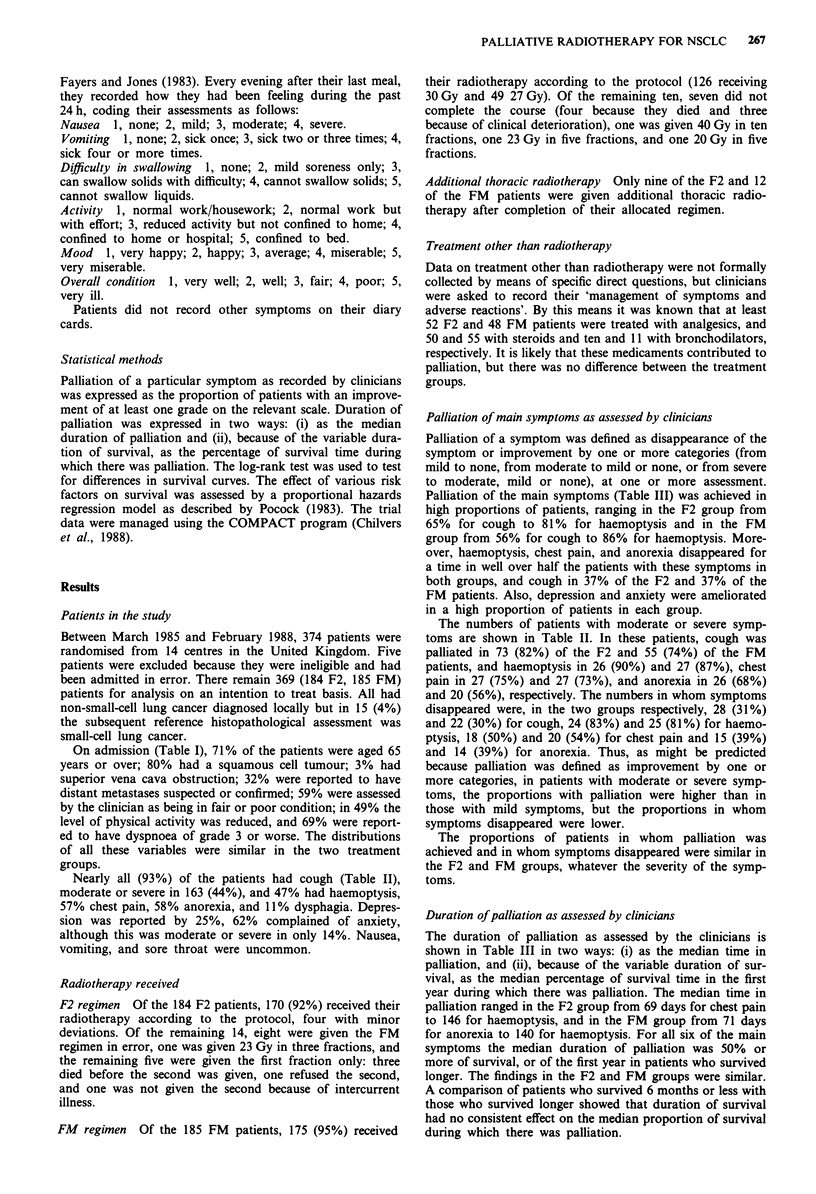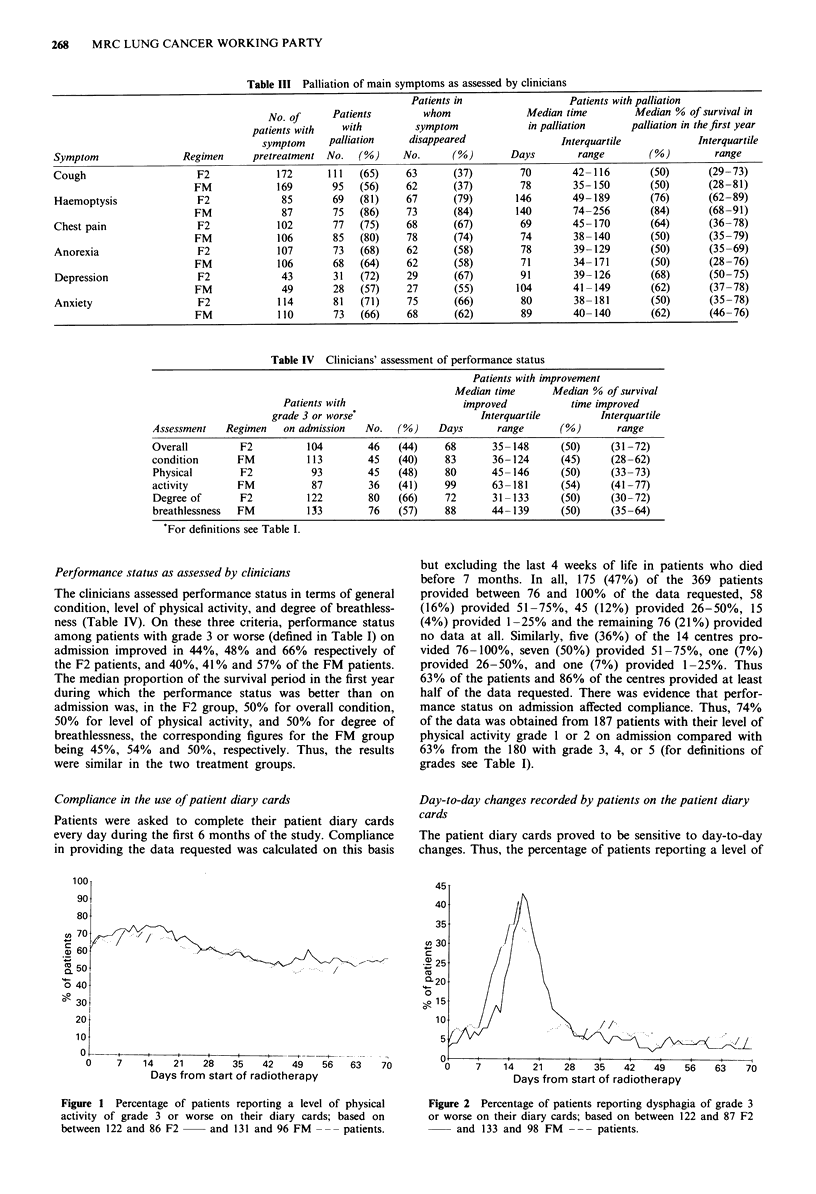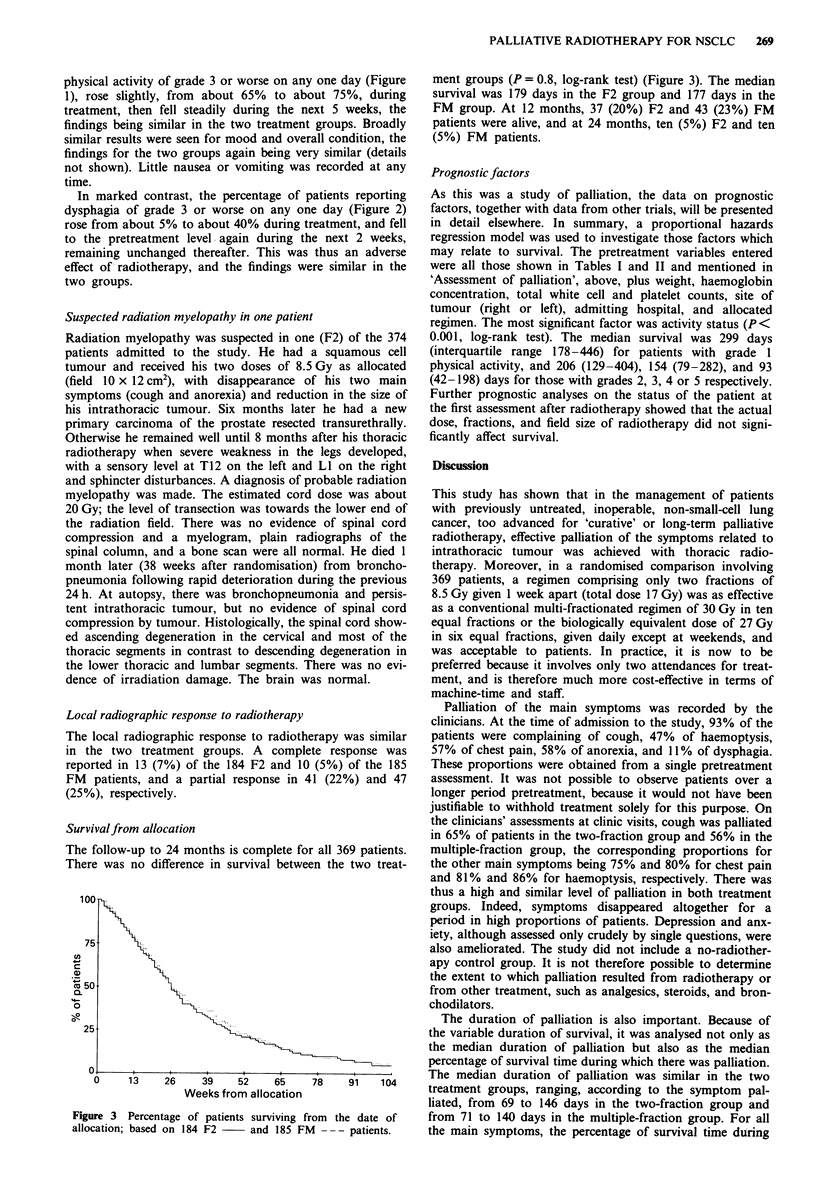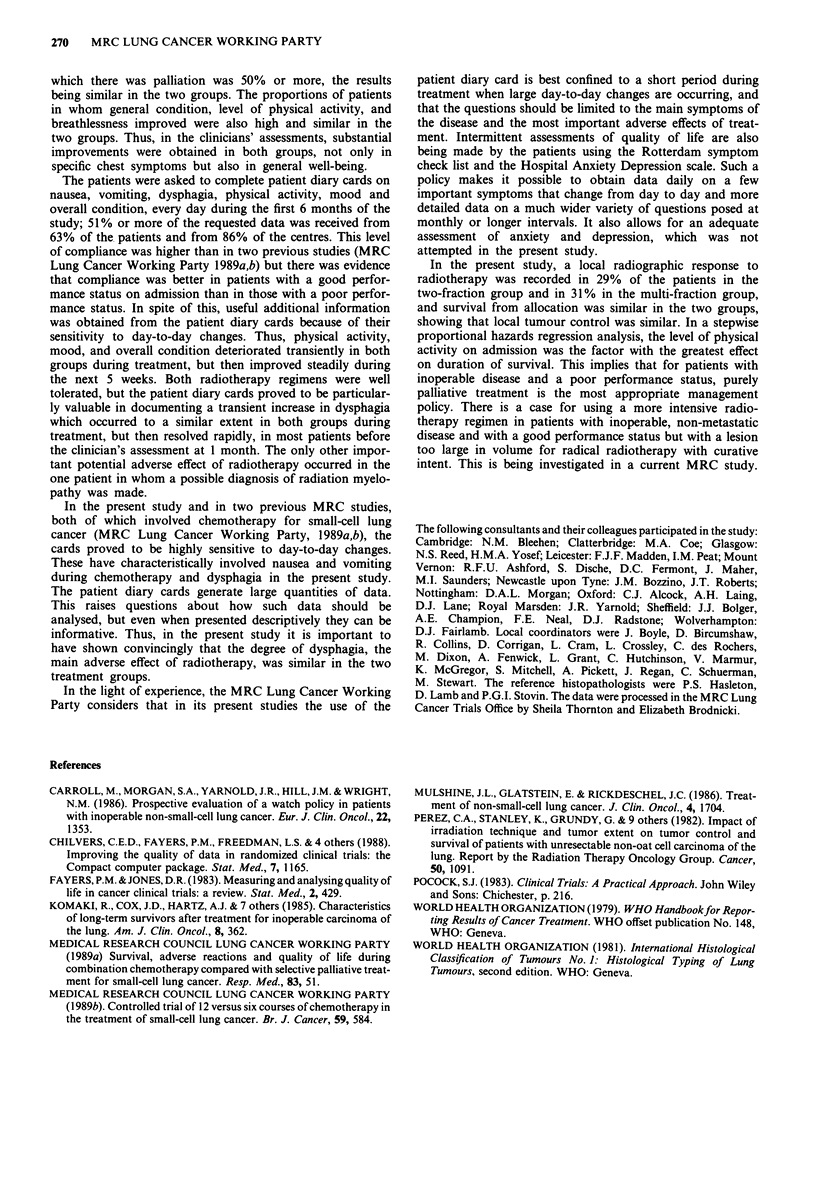# Inoperable non-small-cell lung cancer (NSCLC): a Medical Research Council randomised trial of palliative radiotherapy with two fractions or ten fractions. Report to the Medical Research Council by its Lung Cancer Working Party.

**DOI:** 10.1038/bjc.1991.62

**Published:** 1991-02

**Authors:** 

## Abstract

Two policies of palliative thoracic radiotherapy for non-small-cell lung cancer have been compared in a randomised multicentre controlled trial. A total of 369 patients with inoperable, histologically or cytologically confirmed disease, too advanced for radical 'curative' radiotherapy, and with their main symptoms related to the primary intrathoracic tumour even if metastases were present, were studied. They were allocated at random either to a regimen of 17 Gy given in two fractions of 8.5 Gy 1 week apart (F2 regimen), or to a conventional multifractionated regimen of either 30 Gy in ten fractions or 27 Gy in six fractions (a biologically equivalent dose), given daily except at weekends (FM regimen). On admission, 93% of the patients had cough, 47% haemoptysis, 57% chest pain, 58% anorexia, and 11% dysphagia. As assessed by the clinicians, palliation of the main symptoms was achieved in high proportions of patients ranging in the F2 group from 65% for cough to 81% for haemoptysis and in the FM group from 56% for cough to 86% for haemoptysis. Haemoptysis, chest pain, and anorexia disappeared for a time in well over half the patients with these symptoms, and cough in 37%. For all the main symptoms, the median duration of palliation was 50% or more of survival. Performance status improved in approximately half of the patients with a poor status on admission. All these results were similar in the two treatment groups. As assessed daily by the patients using a diary card, the quality of life deteriorated slightly during treatment but then improved steadily during the next 5 weeks. The proportion of patients with dysphagia increased considerably during treatment, but fell to the pretreatment level during the next 2 weeks. The results were similar in the two groups. Radiation myelopathy was suspected in one (F2) patient. There was no difference in survival between the two groups (log-rank test), the median survival time from the date of allocation being 179 days in the F2 and 177 days in the FM group. In the light of all the findings, the regimen of two fractions of 8.5 Gy given 1 week apart is recommended.


Br. J. Cancer (1991), 63, 265-270 © Macmillan Press Ltd., 1991

### Inoperable non-small-cell lung cancer (NSCLC): a Medical Research Council randomised trial of palliative radiotherapy with two fractions or ten fractions

Report to the Medical Research Council by its Lung Cancer Working Party*

**Summary** Two policies of palliative thoracic radiotherapy for non-small-cell lung cancer have been compared in a randomised multicentre controlled trial. A total of 369 patients with inoperable, histologically or cytologically confirmed disease, too advanced for radical 'curative' radiotherapy, and with their main symptoms related to the primary intrathoracic tumour even if metastases were present, were studied. They were allocated at random either to a regimen of 17 Gy given in two fractions of 8.5 Gy 1 week apart (F2 regimen), or to a conventional multifractionated regimen of either 30 Gy in ten fractions or 27 Gy in six fractions (a biologically equivalent dose), given daily except at weekends (FM regimen). On admission, 93% of the patients had cough, 47% haemoptysis, 57% chest pain, 58% anorexia, and 11% dysphagia. As assessed by the clinicians, palliation of the main symptoms was achieved in high proportions of patients ranging in the F2 group from 65% for cough to 81% for haemoptysis and in the FM group from 56% for cough to 86% for haemoptysis. Haemoptysis, chest pain, and anorexia disappeared for a time in well over half the patients with these symptoms, and cough in 37%. For all the main symptoms, the median duration of palliation was 50% or more of survival. Performance status improved in approximately half of the patients with a poor status on admission. All these results were similar in the two treatment groups.

As assessed daily by the patients using a diary card, the quality of life deteriorated slightly during treatment but then improved steadily during the next 5 weeks. The proportion of patients with dysphagia increased considerably during treatment, but fell to the pretreatment level during the next 2 weeks. The results were similar in the two groups. Radiation myelopathy was suspected in one (F2) patient.

There was no difference in survival between the two groups (log-rank test), the median survival time from the date of allocation being 179 days in the F2 and 177 days in the FM group. In the light of all the findings, the regimen of two fractions of 8.5 Gy given 1 week apart is recommended.

Only a small proportion of patients with inoperable stage I or II non-small-cell lung cancer are cured by primary radiotherapy (Perez *et al.*, 1982; Komaki *et al.*, 1985). The majority present with tumour too advanced for radical radiotherapy, but require palliative treatment for major symptoms related to intrathoracic tumour (Carroll *et al.*, 1986). It is usual to treat such patients, either at first presentation or when significant symptoms develop, with a course of palliative radiotherapy (Mulshine *et al.*, 1986).

The palliative radiotherapy schedule varies considerably in different centres, but in the United Kingdom a typical course would be a dose of 30 Gy given in ten fractions over 2 weeks (Carroll *et al.*, 1986). The present study was conducted to find out whether a shorter course comprising only two fractions of 8.5 Gy (total dose 17 Gy) given 1 week apart gives equally good palliation in the treatment of patients with inoperable non-small-cell lung cancer whose main symptoms are related to intrathoracic tumour. Much of a radiotherapy practice is based on cumulative experience. These two regimens were selected from the experience of members of the working party. If the two-fraction regimen proved effective, it would involve patients in only two attendances for treatment, and would greatly reduce the cost in terms of machine-time and staff.

### Methods

### Eligibility

Patients of either sex and any age were eligible if they had previously untreated, inoperable, histologically or cytologically proved lung cancer of any histological type except small-cell as diagnosed by the local histopathologist; disease considered by the local radiotherapist to be too advanced for 'curative' or long-term palliative radiotherapy; were expected to survive for at least 1 month from admission, and had their main symptoms related to the primary intrathoracic tumour, even if metastases were present. Before a centre entered a patient into the study, local ethics committee approval of the protocol and individual patient consent were obtained.

The diagnoses were made by the histopathologists from the referring centres according to the WHO classification (World Health Organization, 1981). The specimens were later examined by a single reference histopathologist for confirmation of the cell type, and the majority of the specimens by at least one other reference histopathologist.

### *Pretreatment investigations*

The pretreatment investigations included clinical examination, a postero-anterior chest radiograph, measurement of the blood haemoglobin concentration, and total white cell and platelets counts. Evidence of superior vena cava obstruction was recorded when present.

### *Treatment allocation*

Clinicians telephoned the Trials Office and patients were allocated to one or other of two treatment regimens using a minimisation procedure (Pocock, 1983), stratifying for histological type and admitting radiotherapist.

*Two-fraction regimen (F2 regimen)* The patients allocated to the F2 regimen were given megavoltage radiotherapy to a total midline dose of 17 Gy, calculated without air correction, in two fractions of 8.5 Gy 1 week apart.

*Multi-fractionated regimen (FM regimen)* The patients allocated to the FM regimen were given megavoltage radiotherapy to a total midline dose of 30 Gy, calculated without air correction, in ten fractions 5 days per week over 2 weeks, or the biologically equivalent dose of 27 Gy in six fractions, treatment being given daily except at week-ends. The choice of 30 or 27 Gy was left to the individual radiotherapist.

*Prepared on behalf of the participating members by N.M. Bleehen, D.J. Girling, P.M. Fayers, V.R. Aber & R.J. Stephens.
Correspondence: D.J. Girling, MRC Lung Cancer Trials Office, Cardiothoracic Epidemiology Group, Brompton Hospital, Fulham Road, London SW3 6HP, UK.
Received 6 July 1990; and in revised form 13 September 1990.

In both treatment groups, the radiotherapy was delivered through opposing portals to the primary site and mediastinal lymph nodes. The field included the loco-regional tumour volume with a margin of not more than 1 cm, the total area not exceeding 200 cm$^2$. Concurrent steroid administration was recommended for patients with superior vena cava obstruction, but was also permitted in other patients.

*Reports and investigations*

A progress report on each patient was completed monthly up to 12 months and then once every 3 months. These reports included details of the treatment given, the local response to treatment according to the WHO definitions (World Health Organization, 1979), adverse effects encountered, details of any metastases, and the blood haemoglobin concentration and total white cell and platelet counts. At death, the certified cause was reported and, if an autopsy was done, the findings.

*Assessment of palliation by clinicians*

The clinician's assessment of the patient's overall condition, level of physical activity, and degree of breathlessness were recorded at each attendance according to the categories shown in Table I. The clinician also asked the patient about the occurrence and severity, since the last attendance, of the symptoms listed in Table II, and of pain in other sites, nausea, vomiting, sore throat, diarrhoea, and other symptoms, recording the answers as none, mild, moderate, or severe. Details of the management of symptoms and adverse effects of radiotherapy were recorded.

*Daily assessment by patients*

For their first 6 months in the study, the patients completed an MRC patient diary card, similar to that described by

**Table II** Main symptoms on admission as recorded by the clinicians

| Symptom | F2 No. | F2 (%) | FM No. | FM (%) | Total No. | Total (%) |
|---|---|---|---|---|---|---|
| Cough | | | | | | |
| none | 12 | (7) | 15 | (8) | 27 | (7) |
| mild | 83 | (45) | 95 | (52) | 178 | (48) |
| moderate | 80 | (43) | 68 | (37) | 148 | (40) |
| severe | 9 | (5) | 6 | (3) | 15 | (4) |
| Haemoptysis | | | | | | |
| none | 99 | (54) | 97 | (53) | 196 | (53) |
| mild | 56 | (30) | 56 | (30) | 112 | (30) |
| moderate | 24 | (13) | 28 | (15) | 52 | (14) |
| severe | 5 | (3) | 3 | (2) | 8 | (2) |
| Chest pain | | | | | | |
| none | 82 | (45) | 78 | (42) | 160 | (43) |
| mild | 66 | (36) | 69 | (38) | 135 | (37) |
| moderate | 33 | (18) | 33 | (18) | 66 | (18) |
| severe | 3 | (2) | 4 | (2) | 7 | (2) |
| Anorexia | | | | | | |
| none | 75 | (41) | 78 | (42) | 153 | (42) |
| mild | 69 | (38) | 70 | (38) | 139 | (38) |
| moderate | 34 | (19) | 31 | (17) | 65 | (18) |
| severe | 4 | (2) | 5 | (3) | 9 | (2) |
| Dysphagia | | | | | | |
| none | 163 | (89) | 164 | (89) | 327 | (89) |
| mild | 15 | (8) | 16 | (9) | 31 | (8) |
| moderate | 4 | (2) | 4 | (2) | 8 | (2) |
| severe | 1 | (1) | 0 | (0) | 1 | (<1) |
| Depression | | | | | | |
| none | 139 | (76) | 133 | (73) | 272 | (75) |
| mild | 37 | (20) | 41 | (23) | 78 | (21) |
| moderate | 5 | (3) | 7 | (4) | 12 | (3) |
| severe | 1 | (1) | 1 | (1) | 2 | (1) |
| Anxiety | | | | | | |
| none | 68 | (37) | 72 | (40) | 140 | (38) |
| mild | 91 | (50) | 83 | (46) | 174 | (48) |
| moderate | 21 | (12) | 26 | (14) | 47 | (13) |
| severe | 2 | (1) | 1 | (1) | 3 | (1) |

**Table I** General characteristics of the patients on admission

| Characteristic | F2 No. | F2 (%) | FM No. | FM (%) | Total No. | Total (%) |
|---|---|---|---|---|---|---|
| Sex: male | 145 | (79) | 144 | (78) | 289 | (78) |
| Age (years): | | | | | | |
| −44 | 1 | (1) | 1 | (1) | 2 | (1) |
| 45–54 | 10 | (5) | 7 | (4) | 17 | (5) |
| 55–64 | 46 | (25) | 41 | (22) | 87 | (24) |
| 65–74 | 93 | (51) | 90 | (49) | 183 | (50) |
| 75+ | 34 | (18) | 46 | (25) | 80 | (22) |
| Histology (assessed locally) | | | | | | |
| squamous | 147 | (80) | 147 | (79) | 294 | (80) |
| adenocarcinoma | 15 | (8) | 16 | (9) | 31 | (8) |
| large-cell | 14 | (8) | 14 | (8) | 28 | (8) |
| undifferentiated | 5 | (3) | 6 | (3) | 11 | (3) |
| untyped | 3 | (2) | 2 | (1) | 5 | (1) |
| Superior vena cava obstruction present | 4 | (2) | 8 | (4) | 12 | (3) |
| Distant metastases | 59 | (32) | 60 | (32) | 119 | (32) |
| Overall condition: | | | | | | |
| 1. Excellent | 10 | (5) | 8 | (4) | 18 | (5) |
| 2. Good | 70 | (38) | 63 | (34) | 133 | (36) |
| 3. Fair | 84 | (46) | 93 | (51) | 177 | (48) |
| 4. Poor | 20 | (11) | 20 | (11) | 40 | (11) |
| 5. Very poor | 0 | (0) | 0 | (0) | 0 | (0) |
| Level of physical activity: | | | | | | |
| 1. At work or active retirement | 15 | (8) | 14 | (8) | 29 | (8) |
| 2. Full activity but not at work | 75 | (41) | 83 | (45) | 158 | (43) |
| 3. Out and about, but activity restricted | 72 | (39) | 67 | (36) | 139 | (38) |
| 4. Confined to home or hospital | 20 | (11) | 17 | (9) | 37 | (10) |
| 5. Confined to bed | 1 | (1) | 3 | (2) | 4 | (1) |
| Degree of breathlessness: | | | | | | |
| 1. Climbs hills or stairs without dyspnoea | 17 | (9) | 12 | (7) | 29 | (8) |
| 2. Walks any distance on flat without dyspnoea | 45 | (24) | 38 | (21) | 83 | (23) |
| 3. Walks over 100 yards without dyspnoea | 48 | (26) | 67 | (37) | 115 | (31) |
| 4. Dyspnoea on walking 100 yards or less | 53 | (29) | 42 | (23) | 95 | (26) |
| 5. Dyspnoea on mild exertion, e.g. undressing | 21 | (11) | 24 | (13) | 45 | (12) |

Fayers and Jones (1983). Every evening after their last meal, they recorded how they had been feeling during the past 24 h, coding their assessments as follows:
*Nausea* 1, none; 2, mild; 3, moderate; 4, severe.
*Vomiting* 1, none; 2, sick once; 3, sick two or three times; 4, sick four or more times.
*Difficulty in swallowing* 1, none; 2, mild soreness only; 3, can swallow solids with difficulty; 4, cannot swallow solids; 5, cannot swallow liquids.
*Activity* 1, normal work/housework; 2, normal work but with effort; 3, reduced activity but not confined to home; 4, confined to home or hospital; 5, confined to bed.
*Mood* 1, very happy; 2, happy; 3, average; 4, miserable; 5, very miserable.
*Overall condition* 1, very well; 2, well; 3, fair; 4, poor; 5, very ill.

Patients did not record other symptoms on their diary cards.

*Statistical methods*

Palliation of a particular symptom as recorded by clinicians was expressed as the proportion of patients with an improvement of at least one grade on the relevant scale. Duration of palliation was expressed in two ways: (i) as the median duration of palliation and (ii), because of the variable duration of survival, as the percentage of survival time during which there was palliation. The log-rank test was used to test for differences in survival curves. The effect of various risk factors on survival was assessed by a proportional hazards regression model as described by Pocock (1983). The trial data were managed using the COMPACT program (Chilvers *et al.*, 1988).

### Results

*Patients in the study*

Between March 1985 and February 1988, 374 patients were randomised from 14 centres in the United Kingdom. Five patients were excluded because they were ineligible and had been admitted in error. There remain 369 (184 F2, 185 FM) patients for analysis on an intention to treat basis. All had non-small-cell lung cancer diagnosed locally but in 15 (4%) the subsequent reference histopathological assessment was small-cell lung cancer.

On admission (Table I), 71% of the patients were aged 65 years or over; 80% had a squamous cell tumour; 3% had superior vena cava obstruction; 32% were reported to have distant metastases suspected or confirmed; 59% were assessed by the clinician as being in fair or poor condition; in 49% the level of physical activity was reduced, and 69% were reported to have dyspnoea of grade 3 or worse. The distributions of all these variables were similar in the two treatment groups.

Nearly all (93%) of the patients had cough (Table II), moderate or severe in 163 (44%), and 47% had haemoptysis, 57% chest pain, 58% anorexia, and 11% dysphagia. Depression was reported by 25%, 62% complained of anxiety, although this was moderate or severe in only 14%. Nausea, vomiting, and sore throat were uncommon.

*Radiotherapy received*

*F2 regimen* Of the 184 F2 patients, 170 (92%) received their radiotherapy according to the protocol, four with minor deviations. Of the remaining 14, eight were given the FM regimen in error, one was given 23 Gy in three fractions, and the remaining five were given the first fraction only: three died before the second was given, one refused the second, and one was not given the second because of intercurrent illness.

*FM regimen* Of the 185 FM patients, 175 (95%) received their radiotherapy according to the protocol (126 receiving 30 Gy and 49 27 Gy). Of the remaining ten, seven did not complete the course (four because they died and three because of clinical deterioration), one was given 40 Gy in ten fractions, one 23 Gy in five fractions, and one 20 Gy in five fractions.

*Additional thoracic radiotherapy* Only nine of the F2 and 12 of the FM patients were given additional thoracic radiotherapy after completion of their allocated regimen.

*Treatment other than radiotherapy*

Data on treatment other than radiotherapy were not formally collected by means of specific direct questions, but clinicians were asked to record their 'management of symptoms and adverse reactions'. By this means it was known that at least 52 F2 and 48 FM patients were treated with analgesics, and 50 and 55 with steroids and ten and 11 with bronchodilators, respectively. It is likely that these medicaments contributed to palliation, but there was no difference between the treatment groups.

*Palliation of main symptoms as assessed by clinicians*

Palliation of a symptom was defined as disappearance of the symptom or improvement by one or more categories (from mild to none, from moderate to mild or none, or from severe to moderate, mild or none), at one or more assessment. Palliation of the main symptoms (Table III) was achieved in high proportions of patients, ranging in the F2 group from 65% for cough to 81% for haemoptysis and in the FM group from 56% for cough to 86% for haemoptysis. Moreover, haemoptysis, chest pain, and anorexia disappeared for a time in well over half the patients with these symptoms in both groups, and cough in 37% of the F2 and 37% of the FM patients. Also, depression and anxiety were ameliorated in a high proportion of patients in each group.

The numbers of patients with moderate or severe symptoms are shown in Table II. In these patients, cough was palliated in 73 (82%) of the F2 and 55 (74%) of the FM patients, and haemoptysis in 26 (90%) and 27 (87%), chest pain in 27 (75%) and 27 (73%), and anorexia in 26 (68%) and 20 (56%), respectively. The numbers in whom symptoms disappeared were, in the two groups respectively, 28 (31%) and 22 (30%) for cough, 24 (83%) and 25 (81%) for haemoptysis, 18 (50%) and 20 (54%) for chest pain and 15 (39%) and 14 (39%) for anorexia. Thus, as might be predicted because palliation was defined as improvement by one or more categories, in patients with moderate or severe symptoms, the proportions with palliation were higher than in those with mild symptoms, but the proportions in whom symptoms disappeared were lower.

The proportions of patients in whom palliation was achieved and in whom symptoms disappeared were similar in the F2 and FM groups, whatever the severity of the symptoms.

*Duration of palliation as assessed by clinicians*

The duration of palliation as assessed by the clinicians is shown in Table III in two ways: (i) as the median time in palliation, and (ii), because of the variable duration of survival, as the median percentage of survival time in the first year during which there was palliation. The median time in palliation ranged in the F2 group from 69 days for chest pain to 146 for haemoptysis, and in the FM group from 71 days for anorexia to 140 for haemoptysis. For all six of the main symptoms the median duration of palliation was 50% or more of survival, or of the first year in patients who survived longer. The findings in the F2 and FM groups were similar. A comparison of patients who survived 6 months or less with those who survived longer showed that duration of survival had no consistent effect on the median proportion of survival during which there was palliation.

**Table III** Palliation of main symptoms as assessed by clinicians

| Symptom | Regimen | No. of patients with symptom pretreatment | Patients with palliation No. | (%) | Patients in whom symptom disappeared No. | (%) | Patients with palliation: Median time in palliation Days | Interquartile range | Median % of survival in palliation in the first year (%) | Interquartile range |
|---|---|---|---|---|---|---|---|---|---|---|
| Cough | F2 | 172 | 111 | (65) | 63 | (37) | 70 | 42–116 | (50) | (29–73) |
| | FM | 169 | 95 | (56) | 62 | (37) | 78 | 35–150 | (50) | (28–81) |
| Haemoptysis | F2 | 85 | 69 | (81) | 67 | (79) | 146 | 49–189 | (76) | (62–89) |
| | FM | 87 | 75 | (86) | 73 | (84) | 140 | 74–256 | (84) | (68–91) |
| Chest pain | F2 | 102 | 77 | (75) | 68 | (67) | 69 | 45–170 | (64) | (36–78) |
| | FM | 106 | 85 | (80) | 78 | (74) | 74 | 38–140 | (50) | (35–79) |
| Anorexia | F2 | 107 | 73 | (68) | 62 | (58) | 78 | 39–129 | (50) | (35–69) |
| | FM | 106 | 68 | (64) | 62 | (58) | 71 | 34–171 | (50) | (28–76) |
| Depression | F2 | 43 | 31 | (72) | 29 | (67) | 91 | 39–126 | (68) | (50–75) |
| | FM | 49 | 28 | (57) | 27 | (55) | 104 | 41–149 | (62) | (37–78) |
| Anxiety | F2 | 114 | 81 | (71) | 75 | (66) | 80 | 38–181 | (50) | (35–78) |
| | FM | 110 | 73 | (66) | 68 | (62) | 89 | 40–140 | (62) | (46–76) |

**Table IV** Clinicians' assessment of performance status

| Assessment | Regimen | Patients with grade 3 or worse* on admission | Patients with improvement No. | (%) | Median time improved Days | Interquartile range | Median % of survival time improved (%) | Interquartile range |
|---|---|---|---|---|---|---|---|---|
| Overall | F2 | 104 | 46 | (44) | 68 | 35–148 | (50) | (31–72) |
| condition | FM | 113 | 45 | (40) | 83 | 36–124 | (45) | (28–62) |
| Physical | F2 | 93 | 45 | (48) | 80 | 45–146 | (50) | (33–73) |
| activity | FM | 87 | 36 | (41) | 99 | 63–181 | (54) | (41–77) |
| Degree of | F2 | 122 | 80 | (66) | 72 | 31–133 | (50) | (30–72) |
| breathlessness | FM | 133 | 76 | (57) | 88 | 44–139 | (50) | (35–64) |

*For definitions see Table I.

*Performance status as assessed by clinicians*

The clinicians assessed performance status in terms of general condition, level of physical activity, and degree of breathlessness (Table IV). On these three criteria, performance status among patients with grade 3 or worse (defined in Table I) on admission improved in 44%, 48% and 66% respectively of the F2 patients, and 40%, 41% and 57% of the FM patients. The median proportion of the survival period in the first year during which the performance status was better than on admission was, in the F2 group, 50% for overall condition, 50% for level of physical activity, and 50% for degree of breathlessness, the corresponding figures for the FM group being 45%, 54% and 50%, respectively. Thus, the results were similar in the two treatment groups.

*Compliance in the use of patient diary cards*

Patients were asked to complete their patient diary cards every day during the first 6 months of the study. Compliance in providing the data requested was calculated on this basis but excluding the last 4 weeks of life in patients who died before 7 months. In all, 175 (47%) of the 369 patients provided between 76 and 100% of the data requested, 58 (16%) provided 51–75%, 45 (12%) provided 26–50%, 15 (4%) provided 1–25% and the remaining 76 (21%) provided no data at all. Similarly, five (36%) of the 14 centres provided 76–100%, seven (50%) provided 51–75%, one (7%) provided 26–50%, and one (7%) provided 1–25%. Thus 63% of the patients and 86% of the centres provided at least half of the data requested. There was evidence that performance status on admission affected compliance. Thus, 74% of the data was obtained from 187 patients with their level of physical activity grade 1 or 2 on admission compared with 63% from the 180 with grade 3, 4, or 5 (for definitions of grades see Table I).

*Day-to-day changes recorded by patients on the patient diary cards*

The patient diary cards proved to be sensitive to day-to-day changes. Thus, the percentage of patients reporting a level of

**Figure 1** Percentage of patients reporting a level of physical activity of grade 3 or worse on their diary cards; based on between 122 and 86 F2 —— and 131 and 96 FM --- patients.

**Figure 2** Percentage of patients reporting dysphagia of grade 3 or worse on their diary cards; based on between 122 and 87 F2 —— and 133 and 98 FM --- patients.

physical activity of grade 3 or worse on any one day (Figure 1), rose slightly, from about 65% to about 75%, during treatment, then fell steadily during the next 5 weeks, the findings being similar in the two treatment groups. Broadly similar results were seen for mood and overall condition, the findings for the two groups again being very similar (details not shown). Little nausea or vomiting was recorded at any time.

In marked contrast, the percentage of patients reporting dysphagia of grade 3 or worse on any one day (Figure 2) rose from about 5% to about 40% during treatment, and fell to the pretreatment level again during the next 2 weeks, remaining unchanged thereafter. This was thus an adverse effect of radiotherapy, and the findings were similar in the two groups.

*Suspected radiation myelopathy in one patient*

Radiation myelopathy was suspected in one (F2) of the 374 patients admitted to the study. He had a squamous cell tumour and received his two doses of 8.5 Gy as allocated (field 10 × 12 $cm^2$), with disappearance of his two main symptoms (cough and anorexia) and reduction in the size of his intrathoracic tumour. Six months later he had a new primary carcinoma of the prostate resected transurethrally. Otherwise he remained well until 8 months after his thoracic radiotherapy when severe weakness in the legs developed, with a sensory level at T12 on the left and L1 on the right and sphincter disturbances. A diagnosis of probable radiation myelopathy was made. The estimated cord dose was about 20 Gy; the level of transection was towards the lower end of the radiation field. There was no evidence of spinal cord compression and a myelogram, plain radiographs of the spinal column, and a bone scan were all normal. He died 1 month later (38 weeks after randomisation) from bronchopneumonia following rapid deterioration during the previous 24 h. At autopsy, there was bronchopneumonia and persistent intrathoracic tumour, but no evidence of spinal cord compression by tumour. Histologically, the spinal cord showed ascending degeneration in the cervical and most of the thoracic segments in contrast to descending degeneration in the lower thoracic and lumbar segments. There was no evidence of irradiation damage. The brain was normal.

*Local radiographic response to radiotherapy*

The local radiographic response to radiotherapy was similar in the two treatment groups. A complete response was reported in 13 (7%) of the 184 F2 and 10 (5%) of the 185 FM patients, and a partial response in 41 (22%) and 47 (25%), respectively.

*Survival from allocation*

The follow-up to 24 months is complete for all 369 patients. There was no difference in survival between the two treatment groups ($P = 0.8$, log-rank test) (Figure 3). The median survival was 179 days in the F2 group and 177 days in the FM group. At 12 months, 37 (20%) F2 and 43 (23%) FM patients were alive, and at 24 months, ten (5%) F2 and ten (5%) FM patients.

**Figure 3** Percentage of patients surviving from the date of allocation; based on 184 F2 —— and 185 FM --- patients.

*Prognostic factors*

As this was a study of palliation, the data on prognostic factors, together with data from other trials, will be presented in detail elsewhere. In summary, a proportional hazards regression model was used to investigate those factors which may relate to survival. The pretreatment variables entered were all those shown in Tables I and II and mentioned in 'Assessment of palliation', above, plus weight, haemoglobin concentration, total white cell and platelet counts, site of tumour (right or left), admitting hospital, and allocated regimen. The most significant factor was activity status ($P < 0.001$, log-rank test). The median survival was 299 days (interquartile range 178–446) for patients with grade 1 physical activity, and 206 (129–404), 154 (79–282), and 93 (42–198) days for those with grades 2, 3, 4 or 5 respectively. Further prognostic analyses on the status of the patient at the first assessment after radiotherapy showed that the actual dose, fractions, and field size of radiotherapy did not significantly affect survival.

### Discussion

This study has shown that in the management of patients with previously untreated, inoperable, non-small-cell lung cancer, too advanced for 'curative' or long-term palliative radiotherapy, effective palliation of the symptoms related to intrathoracic tumour was achieved with thoracic radiotherapy. Moreover, in a randomised comparison involving 369 patients, a regimen comprising only two fractions of 8.5 Gy given 1 week apart (total dose 17 Gy) was as effective as a conventional multi-fractionated regimen of 30 Gy in ten equal fractions or the biologically equivalent dose of 27 Gy in six equal fractions, given daily except at weekends, and was acceptable to patients. In practice, it is now to be preferred because it involves only two attendances for treatment, and is therefore much more cost-effective in terms of machine-time and staff.

Palliation of the main symptoms was recorded by the clinicians. At the time of admission to the study, 93% of the patients were complaining of cough, 47% of haemoptysis, 57% of chest pain, 58% of anorexia, and 11% of dysphagia. These proportions were obtained from a single pretreatment assessment. It was not possible to observe patients over a longer period pretreatment, because it would not have been justifiable to withhold treatment solely for this purpose. On the clinicians' assessments at clinic visits, cough was palliated in 65% of patients in the two-fraction group and 56% in the multiple-fraction group, the corresponding proportions for the other main symptoms being 75% and 80% for chest pain and 81% and 86% for haemoptysis, respectively. There was thus a high and similar level of palliation in both treatment groups. Indeed, symptoms disappeared altogether for a period in high proportions of patients. Depression and anxiety, although assessed only crudely by single questions, were also ameliorated. The study did not include a no-radiotherapy control group. It is not therefore possible to determine the extent to which palliation resulted from radiotherapy or from other treatment, such as analgesics, steroids, and bronchodilators.

The duration of palliation is also important. Because of the variable duration of survival, it was analysed not only as the median duration of palliation but also as the median percentage of survival time during which there was palliation. The median duration of palliation was similar in the two treatment groups, ranging, according to the symptom palliated, from 69 to 146 days in the two-fraction group and from 71 to 140 days in the multiple-fraction group. For all the main symptoms, the percentage of survival time during which there was palliation was 50% or more, the results being similar in the two groups. The proportions of patients in whom general condition, level of physical activity, and breathlessness improved were also high and similar in the two groups. Thus, in the clinicians' assessments, substantial improvements were obtained in both groups, not only in specific chest symptoms but also in general well-being.

The patients were asked to complete patient diary cards on nausea, vomiting, dysphagia, physical activity, mood and overall condition, every day during the first 6 months of the study; 51% or more of the requested data was received from 63% of the patients and from 86% of the centres. This level of compliance was higher than in two previous studies (MRC Lung Cancer Working Party 1989*a,b*) but there was evidence that compliance was better in patients with a good performance status on admission than in those with a poor performance status. In spite of this, useful additional information was obtained from the patient diary cards because of their sensitivity to day-to-day changes. Thus, physical activity, mood, and overall condition deteriorated transiently in both groups during treatment, but then improved steadily during the next 5 weeks. Both radiotherapy regimens were well tolerated, but the patient diary cards proved to be particularly valuable in documenting a transient increase in dysphagia which occurred to a similar extent in both groups during treatment, but then resolved rapidly, in most patients before the clinician's assessment at 1 month. The only other important potential adverse effect of radiotherapy occurred in the one patient in whom a possible diagnosis of radiation myelopathy was made.

In the present study and in two previous MRC studies, both of which involved chemotherapy for small-cell lung cancer (MRC Lung Cancer Working Party, 1989*a,b*), the cards proved to be highly sensitive to day-to-day changes. These have characteristically involved nausea and vomiting during chemotherapy and dysphagia in the present study. The patient diary cards generate large quantities of data. This raises questions about how such data should be analysed, but even when presented descriptively they can be informative. Thus, in the present study it is important to have shown convincingly that the degree of dysphagia, the main adverse effect of radiotherapy, was similar in the two treatment groups.

In the light of experience, the MRC Lung Cancer Working Party considers that in its present studies the use of the patient diary card is best confined to a short period during treatment when large day-to-day changes are occurring, and that the questions should be limited to the main symptoms of the disease and the most important adverse effects of treatment. Intermittent assessments of quality of life are also being made by the patients using the Rotterdam symptom check list and the Hospital Anxiety Depression scale. Such a policy makes it possible to obtain data daily on a few important symptoms that change from day to day and more detailed data on a much wider variety of questions posed at monthly or longer intervals. It also allows for an adequate assessment of anxiety and depression, which was not attempted in the present study.

In the present study, a local radiographic response to radiotherapy was recorded in 29% of the patients in the two-fraction group and in 31% in the multi-fraction group, and survival from allocation was similar in the two groups, showing that local tumour control was similar. In a stepwise proportional hazards regression analysis, the level of physical activity on admission was the factor with the greatest effect on duration of survival. This implies that for patients with inoperable disease and a poor performance status, purely palliative treatment is the most appropriate management policy. There is a case for using a more intensive radiotherapy regimen in patients with inoperable, non-metastatic disease and with a good performance status but with a lesion too large in volume for radical radiotherapy with curative intent. This is being investigated in a current MRC study.

The following consultants and their colleagues participated in the study: Cambridge: N.M. Bleehen; Clatterbridge: M.A. Coe; Glasgow: N.S. Reed, H.M.A. Yosef; Leicester: F.J.F. Madden, I.M. Peat; Mount Vernon: R.F.U. Ashford, S. Dische, D.C. Fermont, J. Maher, M.I. Saunders; Newcastle upon Tyne: J.M. Bozzino, J.T. Roberts; Nottingham: D.A.L. Morgan; Oxford: C.J. Alcock, A.H. Laing, D.J. Lane; Royal Marsden: J.R. Yarnold; Sheffield: J.J. Bolger, A.E. Champion, F.E. Neal, D.J. Radstone; Wolverhampton: D.J. Fairlamb. Local coordinators were J. Boyle, D. Bircumshaw, R. Collins, D. Corrigan, L. Cram, L. Crossley, C. des Rochers, M. Dixon, A. Fenwick, L. Grant, C. Hutchinson, V. Marmur, K. McGregor, S. Mitchell, A. Pickett, J. Regan, C. Schuerman, M. Stewart. The reference histopathologists were P.S. Hasleton, D. Lamb and P.G.I. Stovin. The data were processed in the MRC Lung Cancer Trials Office by Sheila Thornton and Elizabeth Brodnicki.

### References

CARROLL, M., MORGAN, S.A., YARNOLD, J.R., HILL, J.M. & WRIGHT, N.M. (1986). Prospective evaluation of a watch policy in patients with inoperable non-small-cell lung cancer. *Eur. J. Clin. Oncol.*, **22**, 1353.

CHILVERS, C.E.D., FAYERS, P.M., FREEDMAN, L.S. & 4 others (1988). Improving the quality of data in randomized clinical trials: the Compact computer package. *Stat. Med.*, **7**, 1165.

FAYERS, P.M. & JONES, D.R. (1983). Measuring and analysing quality of life in cancer clinical trials: a review. *Stat. Med.*, **2**, 429.

KOMAKI, R., COX, J.D., HARTZ, A.J. & 7 others (1985). Characteristics of long-term survivors after treatment for inoperable carcinoma of the lung. *Am. J. Clin. Oncol.*, **8**, 362.

MEDICAL RESEARCH COUNCIL LUNG CANCER WORKING PARTY (1989*a*) Survival, adverse reactions and quality of life during combination chemotherapy compared with selective palliative treatment for small-cell lung cancer. *Resp. Med.*, **83**, 51.

MEDICAL RESEARCH COUNCIL LUNG CANCER WORKING PARTY (1989*b*). Controlled trial of 12 versus six courses of chemotherapy in the treatment of small-cell lung cancer. *Br. J. Cancer*, **59**, 584.

MULSHINE, J.L., GLATSTEIN, E. & RICKDESCHEL, J.C. (1986). Treatment of non-small-cell lung cancer. *J. Clin. Oncol.*, **4**, 1704.

PEREZ, C.A., STANLEY, K., GRUNDY, G. & 9 others (1982). Impact of irradiation technique and tumor extent on tumor control and survival of patients with unresectable non-oat cell carcinoma of the lung. Report by the Radiation Therapy Oncology Group. *Cancer*, **50**, 1091.

POCOCK, S.J. (1983). *Clinical Trials: A Practical Approach*. John Wiley and Sons: Chichester, p. 216.

WORLD HEALTH ORGANIZATION (1979). *WHO Handbook for Reporting Results of Cancer Treatment*. WHO offset publication No. 148, WHO: Geneva.

WORLD HEALTH ORGANIZATION (1981). *International Histological Classification of Tumours No. 1: Histological Typing of Lung Tumours*, second edition. WHO: Geneva.